# Exploratory Ising model network analysis of cluster headache: mapping conditional associations across symptoms, triggers, and pain localization

**DOI:** 10.1186/s10194-026-02474-0

**Published:** 2026-07-29

**Authors:** Mansoureh Togha, Omid Kohandel Gargari, Fariborz Khorvash, Mohsen Hoseinnezhad, Elham Jafari, Arya Derakhshesh, Shiva Rahimi, Vahid Shaygannejad, Zhale Salami, Mahdi Badiee-Gavarti, Reza Shokrani Foroushani, Sheida Shaafi, Faraidoon Haghdoost

**Affiliations:** 1 Headache Department, Iranian Center of Neurological Research, Neuroscience Institute, Tehran University of Medical Sciences, Tehran, Iran; 2 Neurology Department, Sina Hospital, School of Medicine, Tehran University of Medical Sciences, Tehran, Iran; 3 Department of Neurology, Isfahan University of Medical Sciences, Isfahan, Iran; 4 Student Research Committee, Hamadan University of Medical Sciences, Hamadan, Iran; 5 Department of Neurology, Kurdistan University of Medical Sciences, Sanandaj, Iran; 6 Isfahan Neurosciences Research Center, Isfahan University of Medical Sciences, Isfahan, Iran; 7 Student Research Committee, School of Medicine, Isfahan University of Medical Sciences, Isfahan, Iran; 8 Neuroscience Research Center, Isfahan University of Medical Sciences, Isfahan, Iran; 9 Department of Neurology, School of Medicine, Tabriz University of Medical Sciences, Tabriz, Iran; 10 The George Institute for Global Health, University of New South Wales, Sydney, Australia

**Keywords:** Cluster headache, Ising model, Network analysis, Symptoms, Headache triggers, Pain localization, Bridge centrality, Binary networks, Registry-based study, Iran

## Abstract

**Background:**

Cluster headache (CH) is a rare and disabling primary headache disorder characterized by severe unilateral pain and cranial autonomic symptoms. Although its diagnostic criteria are well defined, the conditional relationships among symptoms, reported triggers, and pain locations are not well characterized. Network analysis may provide an exploratory framework for examining these co-occurrence patterns.

**Objective:**

To characterize conditional associations among symptoms, reported triggers, and headache regions in patients with CH using Ising model-based network analysis and to evaluate the stability of the estimated edge and centrality patterns.

**Methods:**

A cross-sectional analysis was conducted using data from the Iranian National Cluster Headache Registry. Binary variables indicating the presence or absence of symptoms, reported triggers, and headache regions were analyzed. Separate symptom, trigger, and headache-region networks, together with an integrated network, were estimated using EBIC-regularized Ising models (γ = 0.25) in R (v4.4.3). Strength and bridge-strength point estimates were calculated. Nonparametric and case-dropping bootstrap procedures (1,000–3,000 iterations) were used to assess edge-weight accuracy and correlation stability (CS); CS coefficients below 0.25 were considered insufficient for robust interpretation of centrality rankings.

**Results:**

Data from 321 patients were analyzed. In the symptom network, the largest estimated positive edges included epiphora–runny nose (1.80) and miosis–runny nose (1.05); epiphora and miosis had the highest strength point estimates, but strength centrality did not meet the prespecified stability threshold (CS = 0.206). The trigger network was unstable (edge CS = 0.05; strength CS = 0.00), and trigger edge and centrality rankings were therefore not interpreted. The headache-region network showed stronger stability (edge CS = 0.748; strength CS = 0.361), with a positive neck–occipital association (2.16) and a negative lower-jaw–cheek association (−2.31). In the integrated network, edge stability was moderate (CS = 0.439), whereas strength centrality remained below the interpretation threshold (CS = 0.206); bridge-strength estimates, including runny nose (2.17), were treated as exploratory.

**Conclusion:**

Ising network analysis identified conditional co-occurrence patterns among CH features, with the most robust findings arising from the headache-region network. Symptom centrality, trigger-network estimates, and integrated centrality and bridge rankings require cautious interpretation because of limited stability. These cross-sectional findings are hypothesis-generating and support validation in larger and longitudinal datasets; they do not establish causal mechanisms or clinically actionable treatment targets.

**Supplementary Information:**

The online version contains supplementary material available at 10.1186/s10194-026-02474-0.

## Highlights

- This study provides an exploratory application of Ising network analysis to symptoms, reported triggers, and pain regions in cluster headache.
- The headache-region network showed the strongest stability, including a positive neck–occipital association and a negative lower-jaw–cheek association.
- Symptom and integrated node-strength rankings did not meet the prespecified stability threshold, and the trigger network was highly unstable.
- Bridge-strength estimates in the integrated network are hypothesis-generating because bridge-specific stability was not established and overall strength centrality was unstable.
- The findings describe cross-sectional conditional associations and require larger, longitudinal, and external validation before clinical or treatment-related applications are considered.

**Supplementary Information:**

The online version contains supplementary material available at 10.1186/s10194-026-02474-0.

## Introduction

Cluster headache (CH) is a rare but severely disabling primary headache disorder, predominantly affecting men and characterized by recurrent, unilateral attacks that substantially impair quality of life [1]. Despite its precise clinical definition, CH is inadequately comprehended, with existing evidence indicating a multifactorial pathophysiology that encompasses the trigeminovascular system, hypothalamic dysfunction, neuropeptide imbalances, and the involvement of the autonomic nervous system [2]. Additionally, CH is often underdiagnosed and inadequately managed, with patients frequently experiencing a diagnostic delay exceeding five years, as indicated by a significant U.S. survey [1]. The clinical heterogeneity and delayed recognition highlight the complexity and challenges in understanding the underlying mechanisms of CH.

Conventional methods for diagnosing and analyzing headache disorders, as detailed in the International Classification of Headache Disorders, 3rd edition (ICHD-3), predominantly utilize symptom checklists and categorical criteria [3]. Linear approaches may inadequately capture the complex interactions among clinical features. For instance, symptom presentation and pain localization in cluster headache (CH) differ across phases: dorsal pain typically occurs at onset, whereas pain radiating to regions such as the neck and shoulder is more characteristic in later stages [4]. Behavioral and environmental factors, including smoking, alcohol consumption, and coffee intake, are correlated with the severity and onset age of CH [5, 6]. Furthermore, while CH is predominantly unilateral, side-shifting may occur during or between episodes in some patients [7]. Additionally, dysfunctions of the sympathetic nervous system, such as miosis and partial Horner syndrome, may also present [8–10]. The observed patterns indicate a complex and multifaceted symptom structure that static diagnostic tools cannot fully address, suggesting the necessity for more integrative analytical methodologies.

Network analysis provides a framework for modeling conditional associations among multiple clinical features and for generating hypotheses about their organization [11, 12]. Over recent decades, statistical methods for estimating and evaluating network structures have developed substantially [13–15]. The Ising model, originally developed in statistical physics, has been adapted for binary clinical data [11, 16, 17]. Applications in psychiatric symptom research illustrate its utility for describing patterns of conditional co-occurrence while also emphasizing the need to evaluate edge accuracy and centrality stability before interpreting node rankings [18–20]. This framework is therefore suitable for an exploratory examination of binary CH symptoms, reported triggers, and pain locations.

Building on this foundation, we applied Ising model-based network analysis to examine how symptoms, reported triggers, and pain regions are conditionally associated in patients with cluster headache. The primary objective was to estimate separate and integrated network structures and to evaluate the accuracy and stability of the resulting edge and centrality estimates. We aimed to describe reproducible co-occurrence patterns and generate hypotheses concerning clinical heterogeneity. Because the data are cross-sectional, the analyses were not intended to identify causal mechanisms, treatment targets, or predictors of treatment response.

## Methods

### Study design and data source

This was a cross-sectional analytical study utilizing data collected from the National Cluster Headache Registry of Iran. This prospective, nationwide registry enrolls patients with cluster headache through neurologists working in hospital-based and outpatient neurology centers across Iran, using standardized ICHD-3 diagnostic criteria to ensure case uniformity. Data are captured via two structured online forms—a baseline registration and a follow-up form for each attack (especially for chronic CH)—recording demographics, CH subtype (episodic/chronic), age at onset, attack frequency/duration/intensity (VAS), associated autonomic symptoms, triggers, acute and preventive treatments (including oxygen and triptans), and treatment response; HIT-6 and adverse events are also documented where applicable. To broaden ascertainment beyond clinic attendees, a public screening questionnaire is hosted on the Iranian Headache Association website, with confirmatory contact for suspected cases before inclusion. A three-month pilot at selected centers debugs workflows and data fields; the registry database is routinely backed up, patients are followed by scheduled visits and telephone checks, and participants receive headache diaries to log attacks and responses. The study operates under ethics approval IR.TUMS.NI.REC.1401.047.

### Participants

All adult patients with a confirmed diagnosis of cluster headache enrolled in the registry at the time of data extraction were included. Variables assessed in this study included binary indicators (0 = absent, 1 = present) of headache symptoms, known triggers, and reported headache regions. Inclusion was limited to individuals with sufficient completeness of data across these domains.

### Data preparation

All variables were coded as binary (0 = absent, 1 = present). Before network estimation, candidate variables were screened for insufficient variability, defined as a standard deviation more than 2.5 SD below the mean SD across candidate variables. Potential redundancy was assessed using the redundant() function in the networktools package. After screening, the final analyzed set comprised eight symptoms (epiphora, miosis, ptosis, runny nose, red eye, eye swelling, facial sweating, and ear stiffness), eight reported triggers (stress, sleep deprivation, hunger, hypersleepiness, alcohol, smoking, spices, and heavy food), and seven pain regions (periorbital, temple, parietal, occipital, neck, cheek, and lower jaw). The final reported networks treated each retained node as a distinct registry variable. No feature was excluded during screening.

### Network estimation

Ising networks were estimated separately for symptoms, reported triggers, and headache regions and then for all retained variables combined. Models were estimated with the IsingFit package using nodewise L1-regularized logistic regressions, with model selection based on the Extended Bayesian Information Criterion (EBIC). The EBIC hyperparameter gamma (γ), which controls network sparsity, was set to 0.25 for all models. The AND rule was used so that an edge was retained only when the corresponding interaction was selected in both nodewise regressions.

### Network visualization and centrality

Networks were visualized using the qgraph package with a spring layout. Strength centrality, defined as the sum of the absolute weights of all edges connected to a node, was calculated using centrality_auto() in qgraph. Strength values were treated as descriptive measures of network connectivity and were not equated with causal influence, biological importance, or clinical priority. Closeness and betweenness were not interpreted because these indices are often unstable in clinical network data.

### Stability and accuracy assessment

Edge-weight accuracy was evaluated using nonparametric bootstrap confidence intervals based on 1,000–3,000 resamples. Case-dropping bootstrap procedures in the bootnet package were used to calculate correlation stability (CS) coefficients for edge weights, thresholds, and centrality indices. A CS coefficient of at least 0.25 was considered the minimum for cautious interpretation, whereas values above 0.50 are generally preferable. When CS coefficients were below 0.25, rankings and substantive interpretations were withheld and the corresponding estimates were reported only as exploratory point estimates.

### Bridge centrality (combined network)

In the integrated model, bridge strength was calculated using the bridge() function in the networktools package after defining symptoms, reported triggers, and pain regions as separate communities. Bridge strength summarizes the absolute weights of edges connecting a node to nodes in other communities. Because a separate CS coefficient for bridge strength was not available, bridge rankings were interpreted as exploratory, particularly when overall node-strength centrality was unstable. Cross-sectional bridge edges were not interpreted as causal mechanisms, activation pathways, or treatment targets.

### Software

All analyses were conducted in R (version 4.4.3) using the IsingFit, qgraph, bootnet, and networktools packages. No inferential *p* values were used to declare individual edges significant; interpretation was based on regularized point estimates together with bootstrap accuracy and stability diagnostics.

## Results

### Patient characteristics

A total of 321 patients with cluster headache were included. The mean age was 41.6 ± 12.2 years (range: 17–84), and most patients were male (78.2%). The mean duration of headache disorder was 10.6 ± 10.0 years, indicating substantial variability in disease chronicity.

The periorbital region was the most frequently affected headache location (87.5%), followed by the lower jaw (80.7%) and temple (67.6%). Among associated autonomic symptoms, epiphora (70.4%), red eye (53.9%), runny nose (45.5%), and ptosis (41.7%) were most commonly reported. The most frequent headache triggers were stress (30.8%), sleep changes (29.0%), and sleep deprivation (27.7%). Detailed demographic and clinical characteristics are presented in Table 1.

**Table 1:** Baseline demographic, anthropometric, and clinical characteristics of patients with cluster headache (*N* = 321). Percentages for headache locations, associated symptoms, and triggers may exceed 100% as multiple responses were allowed

Category	Variable	n (%) or Mean ± SD
Demographics	Age, years	41.6 ± 12.2
	Female sex	70 (21.8%)
Anthropometrics	Height, cm	172.3 ± 14.8
	Weight, kg	77.8 ± 14.7
Disease characteristics	Headache duration, years	10.6 ± 10.0
Headache location	Periorbital	281 (87.5%)
	Lower jaw	259 (80.7%)
	Temple	217 (67.6%)
	Parietal	90 (28.0%)
	Cheeks	80 (24.9%)
	Occipital	69 (21.5%)
	Neck	61 (19.0%)
Associated symptoms	Epiphora	226 (70.4%)
	Red eye	173 (53.9%)
	Runny nose	146 (45.5%)
	Ptosis	134 (41.7%)
	Facial diaphoresis	108 (33.6%)
	Eye swelling	65 (20.2%)
	Miosis	60 (18.7%)
	Ear stiffness	42 (13.1%)
Triggers	Stress	99 (30.8%)
	Sleep changes	93 (29.0%)
	Sleep deprivation	89 (27.7%)
	Hunger	56 (17.4%)
	Smoking	45 (14.0%)
	Alcohol	19 (5.9%)
	Heavy food	15 (4.7%)
	Spicy food	14 (4.4%)
	Hypersomnia	12 (3.7%)

### Symptoms network

**Fig. 1:**
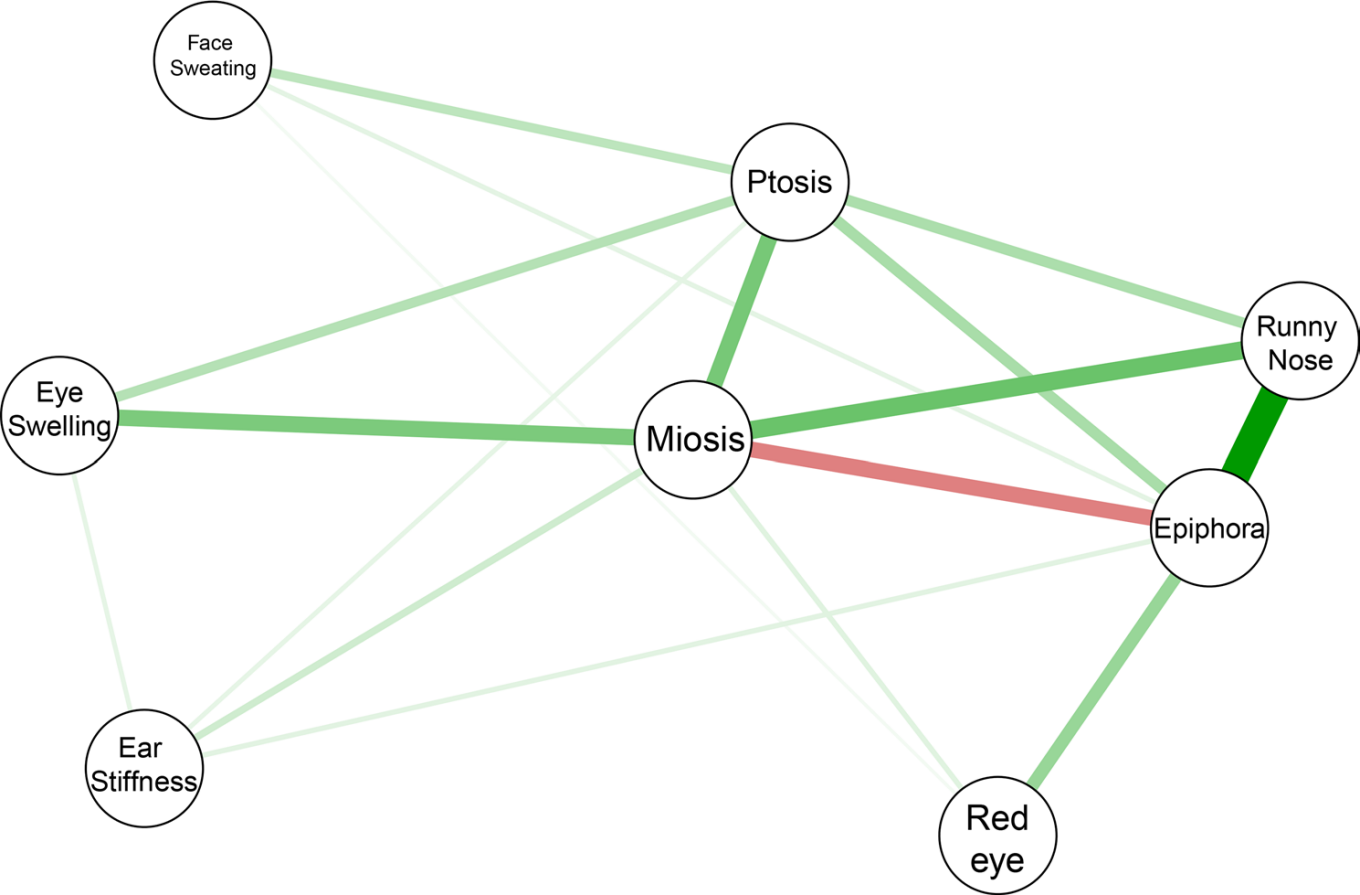
Symptom network structure. This Ising model network displays conditional associations among CH symptoms after adjustment for all other symptoms in the model. Green edges indicate positive associations and red edges indicate negative associations; edge width and saturation reflect the absolute edge weight. The largest estimated positive edges were epiphora–runny nose (1.80), miosis–runny nose (1.05), miosis–ptosis (0.96), and miosis–eye swelling (0.93); the miosis–epiphora edge was negative (−0.90). Edge-weight stability was CS = 0.361, whereas node-strength stability was below threshold (CS = 0.206); strength rankings are therefore descriptive rather than robust. The thickness of each edge is proportional to the magnitude of the estimated edge weight. Numerical edge-weight estimates for all connections are provided in the Supplementary material

The estimated symptom network is shown in Fig. 1. The largest positive edge estimates were observed between epiphora and runny nose (1.80), miosis and runny nose (1.05), miosis and ptosis (0.96), and miosis and eye swelling (0.93). A negative conditional association was estimated between miosis and epiphora (−0.90). These edges represent conditional co-occurrence patterns after adjustment for the other symptoms in the network and should not be interpreted as causal or mutually inhibitory relationships.

Epiphora (4.42) and miosis (4.39) had the highest strength point estimates, followed by runny nose (3.43) and ptosis (3.32), whereas facial sweating (0.74) and ear stiffness (0.90) had lower values. However, because the strength CS coefficient was below the prespecified threshold, these rankings are descriptive and do not provide robust evidence that any symptom is clinically or biologically more important than the others.

Conditional threshold estimates were −2.55 for miosis, −1.89 for ear stiffness, −1.89 for eye swelling, and −1.57 for ptosis. These parameters are model-based conditional intercepts and should not be interpreted as independent prevalence estimates or as the likelihood that a symptom occurs “in isolation.”

The CS coefficient was 0.361 for edge weights and thresholds, supporting cautious interpretation of the larger symptom-edge estimates. In contrast, the CS coefficient for node strength was 0.206, below the minimum threshold of 0.25. Consequently, the relative ordering of symptom strength values was considered unstable and was not used to identify definitive central symptoms (Supplementary Figures 1 and 2).

### Triggers network

**Fig. 2:**
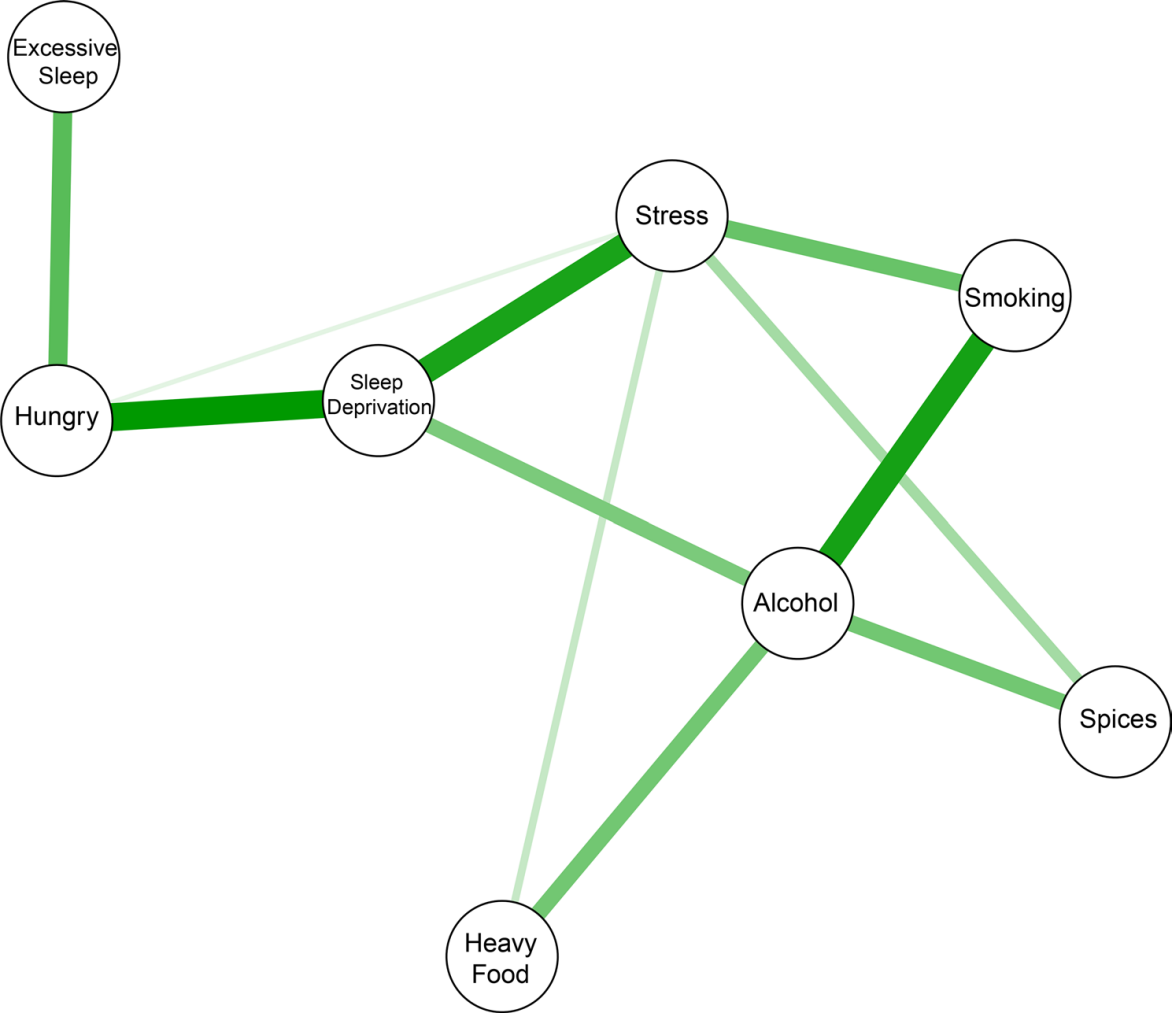
Trigger network structure. This network displays conditional associations among reported CH trigger variables estimated using an Ising model. Each node represents a reported trigger, and edges represent pairwise conditional associations after adjustment for the other triggers. Green edges indicate positive associations and red edges indicate negative associations; edge width and saturation reflect the absolute edge weight. The fitted point estimates are shown for transparency, but the trigger network was not stable (edge CS = 0.05; node-strength CS = 0.00). Consequently, no individual edge, cluster, or central trigger should be interpreted as a robust finding. The thickness of each edge is proportional to the magnitude of the estimated edge weight. Numerical edge-weight estimates for all connections are provided in the Supplementary material

The estimated trigger network is shown in Fig. 2. Point estimates are presented for transparency; however, the bootstrap results indicated that the estimated edge structure was highly sensitive to sampling variation. Accordingly, no individual trigger edge, cluster, or ranking was interpreted as a robust finding.

Strength point estimates were not used to designate “central” or “key” triggers because the case-dropping stability coefficient for strength was 0.00. Apparent differences in connectivity among alcohol, sleep deprivation, stress, and the other trigger nodes should therefore be regarded as sample-specific and non-replicated.

Bootstrap analysis showed a CS coefficient of 0.05 for edge weights and 0.00 for strength centrality, both far below the minimum threshold of 0.25. Thus, the trigger network failed to achieve sufficient stability for substantive interpretation of edge strengths or node importance. Only the threshold estimates showed acceptable stability (CS = 0.673). The trigger network is retained as an exploratory visualization, but no clinical or mechanistic conclusions are drawn from its edge or centrality estimates (Supplementary Figures 3 and 4).

### Headache regions network

**Fig. 3:**
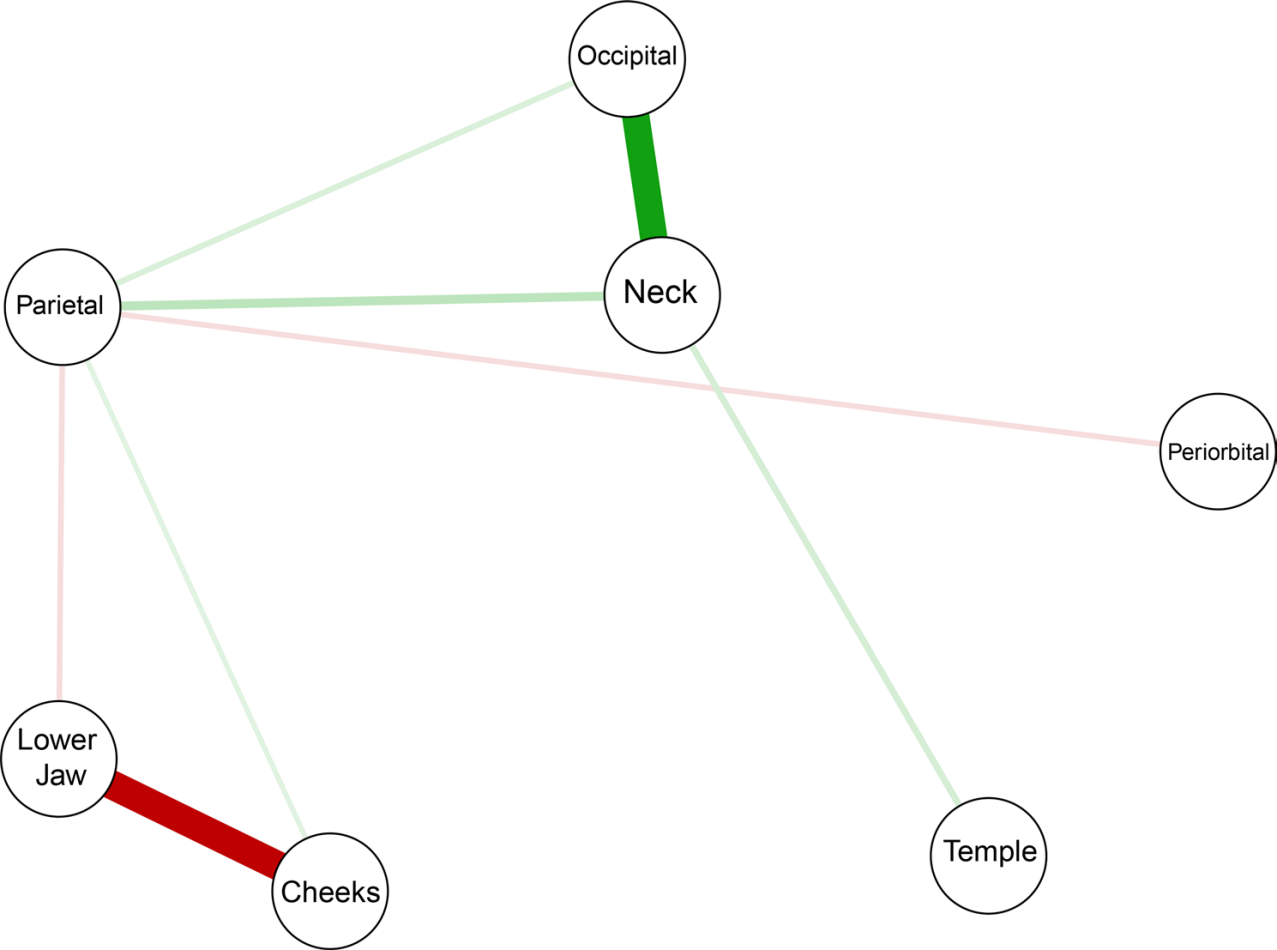
Headache-region network structure. This network displays conditional associations among headache pain locations estimated using an Ising model. Green edges indicate positive associations and red edges indicate negative associations; edge width and saturation reflect the absolute edge weight. The largest positive edge was neck–occipital pain (2.16), and the largest negative edge was lower-jaw–cheek pain (−2.31). Edge-weight stability was high (CS = 0.748), and node-strength stability was acceptable (CS = 0.361), supporting cautious interpretation of the larger pain-location associations. The thickness of each edge is proportional to the magnitude of the estimated edge weight. Numerical edge-weight estimates for all connections are provided in the Supplementary Material

An Ising model was estimated for seven binary headache region variables to examine their co-occurrence structure. The resulting network is displayed in Fig. 3.

The strongest positive conditional association in the headache-region network was observed between neck and occipital pain (2.16). Parietal pain was positively associated with neck (0.60), occipital (0.34), and cheek pain (0.27). The largest negative edge was observed between lower-jaw and cheek pain (−2.31), with a smaller negative edge between parietal and lower-jaw pain (−0.31). These estimates describe conditional co-occurrence and non-co-occurrence patterns rather than anatomic or causal pathways.

Neck pain had the highest strength estimate (3.14), followed by lower-jaw pain (2.62), cheek pain (2.58), and occipital pain (2.49). Periorbital pain (0.31), temple pain (0.38), and parietal pain (1.82) had lower values. Because strength stability met the prespecified minimum threshold in this domain, these relative connectivity patterns may be interpreted with greater confidence than those in the symptom, trigger, or integrated networks, although strength does not imply clinical importance or causality.

Conditional threshold estimates were 2.53 for lower-jaw pain, 2.27 for periorbital pain, −3.00 for neck pain, and −1.75 for occipital pain. These values are conditional model intercepts and are not equivalent to marginal prevalence; observed frequencies are reported separately in Table 1.

Bootstrap analyses supported the relative robustness of the headache-region model. The CS coefficient was 0.748 for edge weights and thresholds and 0.361 for strength centrality. These values exceeded the minimum interpretation threshold and were substantially higher than those observed for the trigger network. The larger headache-region edges and the broad ordering of strength estimates can therefore be interpreted cautiously, while recognizing that they remain cross-sectional conditional associations (Supplementary Figures 5 and 6).

### Combined network

**Fig. 4:**
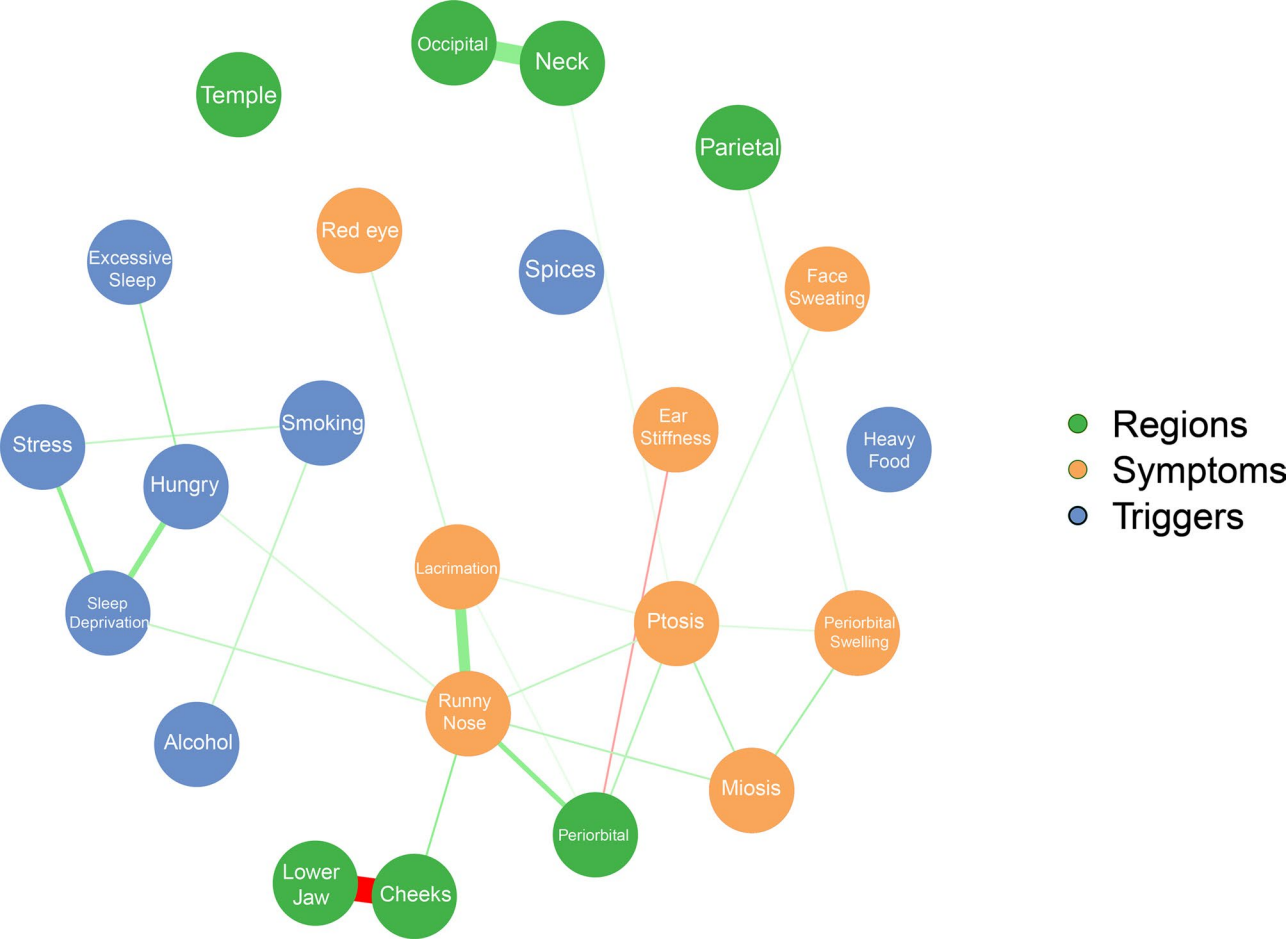
Integrated network of reported triggers, headache regions, and associated symptoms. This network displays conditional associations among the 23 retained CH features. Nodes are grouped into symptom, trigger, and pain-region communities. Green edges indicate positive associations and red edges indicate negative associations; edge width and saturation reflect the absolute edge weight. Selected larger cross-domain point estimates included runny nose–cheek pain (0.68), runny nose–sleep deprivation (0.37), and runny nose–hunger (0.24). Edge-weight stability was CS = 0.439, but node-strength stability was below threshold (CS = 0.206). Bridge-strength point estimates, including runny nose (2.17) and periorbital pain (1.71), are therefore exploratory and should not be interpreted as established pathways or clinically important nodes. The thickness of each edge is proportional to the magnitude of the estimated edge weight. Numerical edge-weight estimates for all connections are provided in the Supplementary material

A comprehensive Ising network was estimated using all 23 retained binary variables: seven headache regions, eight symptoms, and eight reported triggers. The estimated network is shown in Fig. 4. Edge color indicates the sign of the conditional association, and edge width and saturation indicate its absolute magnitude, as defined in the figure legend.

Among the larger point estimates in the integrated network, runny nose was connected with epiphora (1.30), cheek pain (0.68), miosis (0.44), sleep deprivation (0.37), and hunger (0.24). Neck and occipital pain were positively connected (1.85), whereas lower-jaw and cheek pain were negatively connected (−2.29). Ptosis was connected with miosis (0.48), runny nose (0.36), and eye swelling (0.19), and sleep deprivation was connected with stress (0.84). These values describe conditional associations and do not indicate temporal activation or causal influence.

Runny nose (4.28), cheek pain (2.97), lower-jaw pain (2.29), and occipital pain (1.85) had the largest node-strength point estimates in the integrated network, whereas temple pain, spices, heavy food, and alcohol had estimated strength values of 0. Because node-strength stability was below the prespecified threshold, these rankings were treated as descriptive and were not used to identify definitive central features.

Conditional threshold estimates included −3.52 for hypersleepiness, −3.05 for spices, −2.73 for alcohol, 2.53 for lower-jaw pain, 1.60 for periorbital pain, and 0.85 for temple pain. These parameters are conditional intercepts and should not be interpreted as evidence that a feature requires “activation” by another node.

Bridge-strength point estimates were highest for runny nose (2.17), followed by periorbital pain (1.71), cheek pain (0.68), ptosis (0.57), and sleep deprivation (0.37). These estimates identify nodes with relatively larger cross-community connections in the fitted sample. However, a bridge-specific stability coefficient was not available, and overall node-strength stability was below the interpretation threshold; therefore, the bridge ordering is exploratory and does not establish cross-domain pathways, mechanisms, or treatment targets.

Bootstrap analysis showed a CS coefficient of 0.439 for edge weights and 0.748 for thresholds, whereas the CS coefficient for node strength was 0.206, below the minimum threshold of 0.25. Thus, selected larger edges may be discussed cautiously, but node-strength and bridge-strength rankings should be considered unstable and hypothesis-generating. The integrated network does not support definitive claims about node importance (Supplementary Figure 7). Detailed edge weights can be found in the supplementary Excel file.

## Discussion

This exploratory cross-sectional study used Ising models to characterize conditional associations among cluster headache symptoms, reported triggers, and pain locations. The most reproducible findings arose from the headache-region network, particularly the positive neck–occipital and negative lower-jaw–cheek associations. In contrast, symptom centrality did not meet the prespecified stability threshold, the trigger network was highly unstable, and centrality rankings in the integrated network were also unstable. The findings should therefore be interpreted as a description of sample-level co-occurrence patterns rather than as evidence that CH is governed by a validated dynamic system or that specific nodes are clinically actionable.

The trigger network did not achieve adequate stability. Edge-weight stability was 0.05 and strength stability was 0.00, indicating that small changes in the sample could substantially alter both the estimated connections and the ordering of node strength. We therefore do not interpret the apparent trigger clusters, rank alcohol, sleep deprivation, or stress by network importance, or infer shared biological mechanisms from this domain. The trigger analysis is retained to transparently report the attempted model and its failure to produce stable estimates.

Pain-location patterns were more stable. Neck and occipital pain showed a strong positive conditional association, whereas lower-jaw and cheek pain showed a strong negative conditional association. In the integrated model, runny nose, periorbital pain, cheek pain, ptosis, and sleep deprivation had the largest bridge-strength point estimates. However, because integrated node-strength stability was below threshold and bridge-specific stability was not established, these bridge estimates should be viewed only as hypotheses for future validation rather than as established connectors, phenotypes, or guides to personalized management.

The symptom network showed several conditional associations among oculofacial autonomic features, including miosis, ptosis, runny nose, eye swelling, and epiphora. Such clustering is clinically plausible given the involvement of trigeminal–autonomic pathways in CH [3, 21, 22]. However, epiphora and miosis had the largest strength point estimates only in the fitted sample; the strength CS coefficient of 0.206 indicates that their relative ranking is not robust. Similarly, the negative miosis–epiphora edge should not be interpreted as evidence of opposing autonomic mechanisms, because network edges in this cross-sectional model represent conditional statistical associations and may be affected by sampling variability, diagnostic selection, and correlated reporting. Prior clinical studies describe the prominence of cranial autonomic symptoms in CH [1, 23], but the present analysis does not establish that any individual symptom is a biological driver.

Reported triggers remain clinically relevant in CH, and previous studies have frequently described alcohol and sleep disturbance among patient-reported precipitants [1, 24–26]. Nevertheless, the present trigger network cannot provide reliable evidence about how these triggers interrelate. Given the very low stability coefficients, the apparent edges and clusters could change markedly under resampling. We therefore avoid using this model to infer a trigger hierarchy, hypothalamic activation, sympathetic mechanisms, or pathways linking particular triggers to attacks. Larger samples with more balanced trigger endorsement and external replication are needed before network-level trigger findings can be interpreted.

The headache-region network provided the strongest statistical support in this study. The neck–occipital edge (2.16) was stable and is compatible with clinical reports that neck or occipital pain can accompany CH attacks [4, 23]. The negative lower-jaw–cheek edge (−2.31) indicates reduced conditional co-occurrence in this sample, but it does not by itself demonstrate restriction to a specific trigeminal division or establish an anatomic mechanism. Previous studies have emphasized the predominantly unilateral orbital or periorbital distribution of CH pain and its potential radiation to other regions [1, 27]. The relatively high edge CS coefficient (0.748) and acceptable strength CS coefficient (0.361) support cautious interpretation of these pain-location patterns, while the cross-sectional design still precludes temporal or causal conclusions.

In the integrated network, runny nose had the largest bridge-strength point estimate (2.17), followed by periorbital pain (1.71), cheek pain (0.68), ptosis (0.57), and sleep deprivation (0.37). These values indicate relatively larger fitted connections across the predefined symptom, trigger, and pain-region communities. They should not be interpreted as evidence that activation of one feature propagates through the system or precipitates an attack. Overall node-strength stability was below threshold (CS = 0.206), and no bridge-specific CS coefficient was available. The bridge ordering is therefore exploratory and requires independent replication before it can inform phenotyping, prognosis, or treatment research.

A strength of this study is the application of an Ising model, a method originating in statistical physics, to a national registry of a relatively rare headache disorder. Estimating separate domain-specific networks and an integrated network allowed different patterns of conditional association to be examined. Importantly, the bootstrap analyses did not merely support interpretation; they also identified domains in which the estimates were too unstable for substantive conclusions. This explicit distinction between more stable and less stable results improves transparency and helps define priorities for replication.

An important limitation is that several autonomic symptoms in the network are also components of the ICHD-3 diagnostic framework for cluster headache [3]. Because all participants were selected on the basis of a CH diagnosis, criterion-related co-occurrence, diagnostic enrichment, and correlated symptom reporting may create or strengthen associations among these nodes. Consequently, the symptom network cannot distinguish direct biological coupling from patterns induced by case definition or ascertainment. Although the network quantifies conditional associations among retained variables, this does not remove the influence of diagnostic selection. Comparisons with independently ascertained cohorts, other headache disorders, and repeated within-person attack data will be needed to separate disease-specific organization from criterion-based co-occurrence.

Several additional limitations should be considered. The cross-sectional design precludes inference about temporal ordering, symptom activation, prognosis, or treatment response. Binary coding does not capture frequency, severity, timing, or within-person variation. The sample size was limited relative to the number and distribution of variables, and no formal network power calculation was performed. Most importantly, symptom strength and integrated strength did not meet the minimum stability threshold, and the trigger network showed near-zero stability. Bootstrap procedures quantify these limitations but do not overcome them. Accordingly, unstable centrality and bridge rankings should not be treated as robust clinical findings. The national registry is a strength, but external validation in larger and more diverse cohorts remains necessary.

Future studies should use larger, externally validated samples and repeated longitudinal assessments across attacks and disease phases. More granular measures of symptom severity, timing, trigger exposure, and pain spread may improve estimation and allow temporal network approaches. Treatment-response, prognostic, imaging, biomarker, or wearable data could be incorporated prospectively to test whether reproducible network features add predictive value; such clinical utility cannot be inferred from the present study. Side shifting was not included because of limited data and may be evaluated in future work [28]. Comparisons with migraine and other trigeminal autonomic cephalalgias may also help determine which associations are specific to CH. These steps are required before network features can be considered for phenotyping or individualized management.

## Conclusion

This exploratory Ising model analysis identified conditional associations among symptoms, reported triggers, and pain regions in patients with cluster headache. The headache-region network, particularly the neck–occipital and lower-jaw–cheek associations, showed the strongest stability. In contrast, symptom and integrated centrality rankings were unstable, and the trigger network did not support substantive edge or centrality interpretation. Bridge-strength estimates, including those for runny nose and periorbital pain, should therefore be considered hypothesis-generating. The present cross-sectional findings do not identify causal mechanisms or treatment targets and require replication in larger, longitudinal, and externally validated datasets.

## Electronic supplementary material

Below is the link to the electronic supplementary material.

Supplementary Material 1: Bootstrapped Edge-Weight Accuracy in the Symptom Network. Red points represent edge weights estimated in the original sample, black points represent mean bootstrap estimates, and grey intervals represent 95% bootstrap confidence intervals. Narrower intervals indicate greater precision. The edge-weight CS coefficient was 0.361; uncertainty should be considered when interpreting individual edges.

Supplementary Material 2: Case-Dropping Bootstrap Stability of Node Strength in the Symptom Network. The x-axis indicates the proportion of cases retained, and the y-axis indicates the correlation between strength estimates from subsetted networks and the original network. The line shows the mean correlation and the shaded area shows the 95% interval. The node-strength CS coefficient was 0.206, below the minimum threshold of 0.25; symptom strength rankings are therefore unstable and should be interpreted descriptively.

Supplementary Material 3: Bootstrapped Edge-Weight Accuracy in the Trigger Network. Red points represent original-sample edge weights, black points represent mean bootstrap estimates, and grey intervals represent 95% bootstrap confidence intervals. The edge-weight CS coefficient was 0.05, indicating that the trigger-edge structure was highly sensitive to sampling variation. The plot should not be interpreted as demonstrating robust trigger associations.

Supplementary Material 4: Case-Dropping Bootstrap Stability of Node Strength in the Trigger Network. The x-axis shows the proportion of cases retained, and the y-axis shows the correlation between subsetted and original-sample strength estimates. The line represents the mean correlation and the shaded area the 95% interval. The node-strength CS coefficient was 0.00, demonstrating that strength rankings in the trigger network were not stable and should not be interpreted.

Supplementary Material 5: Bootstrapped Edge-Weight Accuracy for the Headache Region Network. Red points represent original-sample edge weights, black points represent mean bootstrap estimates, and grey intervals represent 95% bootstrap confidence intervals. Narrower intervals indicate greater precision. The edge-weight CS coefficient was 0.748, supporting the relative stability of the headache-region edge structure.

Supplementary Material 6: Case-Dropping Bootstrap Stability of Node Strength in the Headache Region Network. The x-axis shows the proportion of cases retained, and the y-axis shows the correlation between strength estimates in subsetted networks and the original network. The solid line represents the mean correlation and the shaded area the 95% interval. The node-strength CS coefficient was 0.361, exceeding the minimum threshold of 0.25 and supporting cautious interpretation of the broad strength ordering in the headache-region network.

Supplementary Material 7: Case-Dropping Bootstrap Stability of Node Strength in the Integrated Network. The x-axis represents the proportion of cases retained, and the y-axis shows the correlation between node-strength estimates in subsetted networks and the original network. The solid line indicates the mean correlation, and the shaded area indicates the 95% interval. The node-strength CS coefficient was 0.206, below the minimum threshold of 0.25. Thus, node-strength rankings in the integrated network are unstable and should not be interpreted as remaining robust after case removal.

Supplementary Material 8

## Data Availability

No datasets were generated or analysed during the current study.
