# Supplementary figures and images for: Exploratory Ising model network analysis of cluster headache: mapping conditional associations across symptoms, triggers, and pain localization
Source: J Headache Pain. 2026 Jul 29;27(1):196. doi: 10.1186/s10194-026-02474-0 (PMC13430735; doi:10.1186/s10194-026-02474-0)

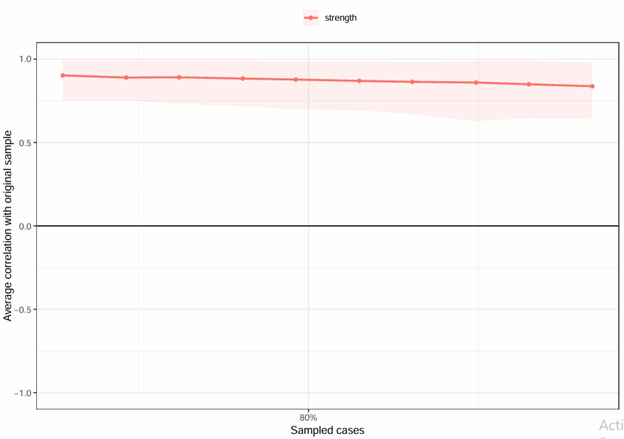

Supplement: Supplementary file 2 — Supplementary Material 2: Case-Dropping Bootstrap Stability of Node Strength in the Symptom Network. The x-axis indicates the proportion of cases retained, and the y-axis indicates the correlation between strength estimates from subsetted networks and the original network. The line shows the mean correlation and the shaded area shows the 95% interval. The node-strength CS coefficient was 0.206, below the minimum threshold of 0.25; symptom strength rankings are therefore unstable and should be interpreted descriptively. [file 10194_2026_2474_MOESM2_ESM.png]

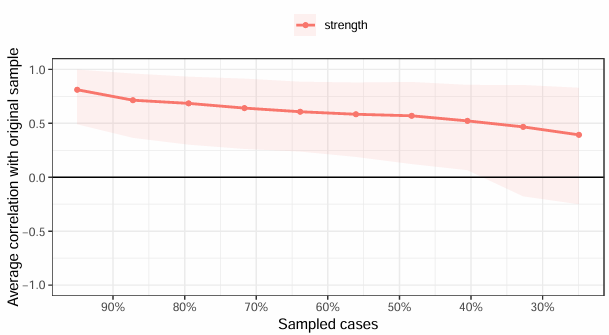

Supplement: Supplementary file 4 — Supplementary Material 4: Case-Dropping Bootstrap Stability of Node Strength in the Trigger Network. The x-axis shows the proportion of cases retained, and the y-axis shows the correlation between subsetted and original-sample strength estimates. The line represents the mean correlation and the shaded area the 95% interval. The node-strength CS coefficient was 0.00, demonstrating that strength rankings in the trigger network were not stable and should not be interpreted. [file 10194_2026_2474_MOESM4_ESM.png]

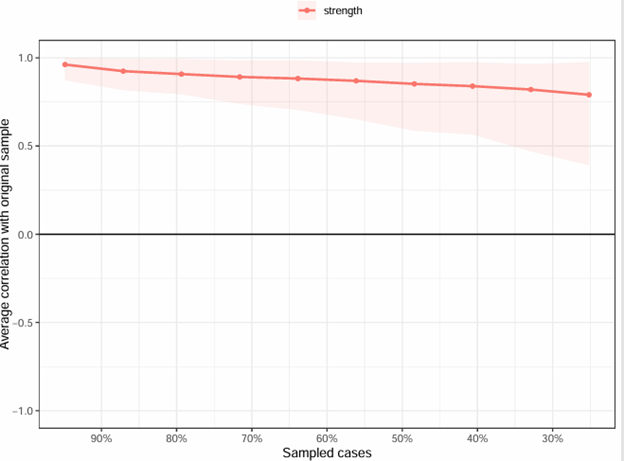

Supplement: Supplementary file 6 — Supplementary Material 6: Case-Dropping Bootstrap Stability of Node Strength in the Headache Region Network. The x-axis shows the proportion of cases retained, and the y-axis shows the correlation between strength estimates in subsetted networks and the original network. The solid line represents the mean correlation and the shaded area the 95% interval. The node-strength CS coefficient was 0.361, exceeding the minimum threshold of 0.25 and supporting cautious interpretation of the broad strength ordering in the headache-region network. [file 10194_2026_2474_MOESM6_ESM.png]

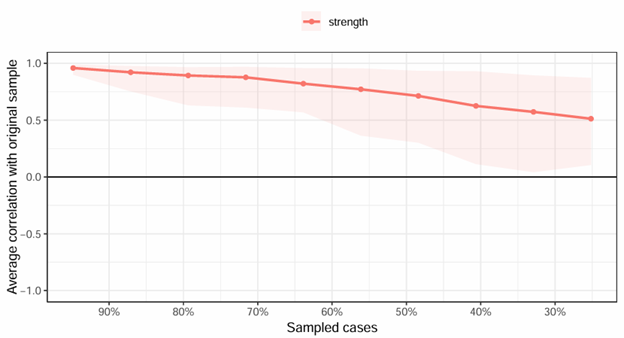

Supplement: Supplementary file 7 — Supplementary Material 7: Case-Dropping Bootstrap Stability of Node Strength in the Integrated Network. The x-axis represents the proportion of cases retained, and the y-axis shows the correlation between node-strength estimates in subsetted networks and the original network. The solid line indicates the mean correlation, and the shaded area indicates the 95% interval. The node-strength CS coefficient was 0.206, below the minimum threshold of 0.25. Thus, node-strength rankings in the integrated network are unstable and should not be interpreted as remaining robust after case removal. [file 10194_2026_2474_MOESM7_ESM.png]
